# A polyketide synthase gene cluster required for pathogenicity of *Pseudocercospora fijiensis* on banana

**DOI:** 10.1371/journal.pone.0258981

**Published:** 2021-10-27

**Authors:** Elizabeth Thomas, Roslyn D. Noar, Margaret E. Daub

**Affiliations:** 1 Department of Plant and Microbial Biology, North Carolina State University, Raleigh, NC, United States of America; 2 NSF Center for Integrated Pest Management, North Carolina State University, Raleigh, NC, United States of America; University of Nebraska-Lincoln, UNITED STATES

## Abstract

*Pseudocercospora fijiensis* is the causal agent of the highly destructive black Sigatoka disease of banana. Previous research has focused on polyketide synthase gene clusters in the fungus, given the importance of polyketide pathways in related plant pathogenic fungi. A time course study of expression of the previously identified *PKS7-1*, *PKS8-2*, and *PKS10-2* gene clusters showed high expression of all three PKS genes and the associated clustered genes in infected banana plants from 2 weeks post-inoculation through 9 weeks. Engineered transformants silenced for *PKS8-2* and *PKS10-2* were developed and tested for pathogenicity. Inoculation of banana plants with silencing transformants for *PKS10-2* showed significant reduction in disease symptoms and severity that correlated with the degree of silencing in the conidia used for inoculation, supporting a critical role for PKS10-2 in disease development. Unlike *PKS10-2*, a clear role for *PKS8-2* could not be determined. Two of four *PKS8-2* silencing transformants showed reduced disease development, but disease did not correlate with the degree of *PKS8-2* silencing in the transformants. Overall, the degree of silencing obtained for the *PKS8-2* transformants was less than that obtained for the *PKS10-2* transformants, which may have limited the utility of the silencing strategy to identify a role for *PKS8-2* in disease. Orthologous *PKS10-2* clusters had previously been identified in the related banana pathogens *Pseudocercospora musae* and *Pseudocercospora eumusae*. Genome analysis identified orthologous gene clusters to that of *PKS10-2* in the newly sequenced genomes of *Pseudocercospora fuligena* and *Pseudocercospora cruenta*, pathogens of tomato and cowpea, respectively. Our results support an important role for the PKS10-2 polyketide pathway in pathogenicity of *Pseudocercospora fijiensis*, and suggest a possible role for this pathway in disease development by other *Pseudocercospora* species.

## Introduction

Banana (including *Musa* species of the common “dessert” bananas as well as cooking bananas and plantains) is one of the world’s most important food crops, grown in tropical and subtropical regions in over 100 countries [1, 2]. Black Sigatoka (also called black leaf streak), caused by the fungus *Pseudocercospora fijiensis* (formerly *Mycosphaerella fijiensis*), is one of the most devastating diseases of banana worldwide [3, 4]. The fungus penetrates the leaves of banana through the stomata, and infection results in necrotic streaking and extensive blighting of leaf tissue, resulting in loss of photosynthetic capacity as well as premature ripening of the banana fruit. The importance of the disease has led to extensive studies to characterize host-pathogen interactions, including sequencing of the *P*. *fijiensis* and banana genomes [5–9]. Significant progress has been made in characterizing defense responses in resistant versus susceptible banana cultivars [8, 10–12]. Some studies have led to the identification and characterization of *P*. *fijiensis* effectors and signaling pathways involved in the *Musa* spp.-*P*. *fijiensis* interaction [13–15]. Other studies have documented a role for the osmotic stress response in virulence of *P*. *fijiensis* [16], and have characterized cell wall protein differences between *P*. *fijiensis* strains that differ in virulence [17].

Our interests have focused on the pathogenicity mechanisms of *P*. *fijiensis*. In a previous study we used transcriptome sequencing to compare *P*. *fijiensis* gene expression in infected leaf tissue with cultures grown in artificial media [18]. This analysis identified a variety of putative pathogenicity genes including those encoding cytochrome P450s, short-chain dehydrogenases, oxidoreductases, salicylate hydroxylase-like proteins, and hydrophobic surface binding proteins, among others. Our main interest has been to identify and characterize possible toxin pathways, as *P*. *fijiensis* is related to several genera of plant pathogenic fungi that are known to produce damaging toxins involved in disease development. The most studied of the related toxin-producing plant pathogenic fungi are species of *Cercospora*, a large genus of fungi that cause serious leaf blighting diseases world-wide on a wide diversity of hosts [19]. *Cercospora* species produce a light-activated toxin, cercosporin, which damages host cells through the production of singlet oxygen (^1^O_2_), a highly reactive species of active oxygen that damages the membranes of host plants, leading to cell death and colonization by the fungus [20–22]. The critical role of cercosporin in disease development in soybean, sugar beet, maize, and tobacco has been documented via studies with cercosporin-deficient mutants of *Cercospora kikuchii*, *Cercospora beticola*, *Cercospora zeae-maydis*, and *Cercospora nicotianae* [23–28]. Cercosporin’s core role in disease has also been confirmed by the recent engineering of tobacco for *Cercospora* disease resistance via both the expression of *C*. *nicotianae* cercosporin-resistance genes and by silencing of the cercosporin biosynthetic pathway [29]. Documentation of the importance of light in disease development in sugar beet and coffee has also supported the critical role of cercosporin in disease [30, 31]. The role of a similar light-activated toxin in black Sigatoka has been hypothesized, as disease symptoms are reduced under shade [32, 33].

Cercosporin is a perylenequinone produced through a polyketide synthase (PKS) pathway, thus previous work in the laboratory has focused on polyketide synthase gene clusters in *P*. *fijiensis* [34–36]. We conducted bioinformatics analyses that identified seven PKS genes (*PKS2-1*, *PKS7-1*, *PKS8-1*, *PKS8-2*, *PKS8-4*, *PKS10-1*, and *PKS10-2*) and one hybrid PKS-nonribosomal peptide synthetase (NRPS) gene (*Hybrid8-3*) in the *P*. *fijiensis* genome [34]. Phylogenetic analysis did not identify a homolog of the PKS enzyme in the cercosporin pathway, but did identify homologs of PKS enzymes in several known PKS pathways such as those encoding melanin, alternapyrone, fumonisin, and solanapyrone [34]. Each of the *P*. *fijiensis* PKS genes was clustered with genes encoding enzymes commonly found in PKS pathways including NRPSs, methyltransferases, oxidoreductases, cytochrome P450s, and transporters. We conducted RNA-Seq analysis of the PKSs and clustered genes in infected leaves at 6 weeks post-inoculation as compared to the fungus grown *in vitro* [34]. This analysis confirmed predictions of the genes that make up each PKS cluster, and also demonstrated significant induction of gene expression for several clusters during disease development.

We have been working to further characterize the *P*. *fijiensis* PKS clusters, focusing on those that are expressed during disease development. Early work had suggested that melanin shunt metabolites might play a role as toxins in disease development [37, 38]. Beltran-Garcia and co-workers documented production of singlet oxygen (^1^O_2_) by *P*. *fijiensis*-produced melanin, and they demonstrated a positive correlation between the presence of melanin in infected leaves and the stage of infection [39], suggesting a role in disease. Other work, however, did not identify differences in disease development in banana inoculated with melanin PKS disruption mutants [4]. We identified a PKS (PKS10-1) that forms a clade with known PKS enzymes in melanin pathways from diverse fungi [34]. Our work found that *PKS10-1* and its clustered genes showed lower expression in infected banana than in culture, however, suggesting a limited role for melanin in disease [34]. Thus, the precise role of melanin in black Sigatoka disease development is not yet known.

We then went on to characterize the *PKS8-1*, *PKS8-4*, and *Hybrid8-3* clusters. Phylogenetic analysis identified PKS8-1 in a clade with PKS enzymes in the related banana pathogens *Pseudocercospora musae* and *Pseudocercospora eumusae* and with enzymes in the monodictyphenone pathway in *Aspergillus nidulans* and the cladofulvin pathway in *Cladosporium fulvum* [35]. An analysis of the clustered genes in *P*. *fijiensis* identified almost identical clusters in *P*. *musae* and *P*. *eumusae*, suggesting a possible role in pathogenicity in Sigatoka diseases of banana. However, there were significant differences in the clustered genes between the *Pseudocercospora* clusters and the monodictyphenone and cladofulvin clusters, suggesting production of different metabolic products. A time course study of expression of *PKS8-1* showed high expression as early as 2 weeks after inoculation and continuing through 9 weeks. Four of eight of its clustered genes were also highly expressed. The role of the *PKS8-1* pathway in disease development was tested by production of overexpression mutants via constitutive expression of a transcription factor within the cluster. This strategy did not result in upregulation of the pathway over the normal high expression of the cluster genes *in planta*, thus no conclusive evidence for a role for the *PKS8-1* cluster was obtained.

Characterization of the *PKS8-4* and *Hybrid8-3* clusters identified them as playing a role in the sexual reproductive cycle of *P*. *fijiensis*, an important discovery as the plant-to-plant spread of the fungus is primarily through ascospores [36]. Phylogenetic analysis identified similarities to polyketide synthases involved in sexual reproduction in *Neurospora crassa* and *Sordaria macrospora* [40]. *P*. *fijiensis* PKS8-4:GFP fusion transformants were generated, and they documented expression of GFP in spermagonia in mating cultures and in leaf substomatal cavities, confirming a role in the sexual reproductive cycle.

Here we report characterization of three remaining PKS gene clusters that are of interest due to their expression during disease development by *P*. *fijiensis* [34]. We conducted a time course of gene expression in the *PKS7-1*, *PKS8-2*, and *PKS10-2* clusters during black Sigatoka symptom development, documenting upregulation throughout the disease cycle. Transformants silenced for the *PKS* genes in the *PKS8-2* and *PKS10-2* clusters were developed, and tested for pathogenicity on banana. Here we show that transformants silenced for *PKS10-2* cluster are significantly less pathogenic, supporting a role for this cluster in black Sigatoka disease.

## Results

### Time course of expression of PKS gene clusters during disease development

**Fig 1:**
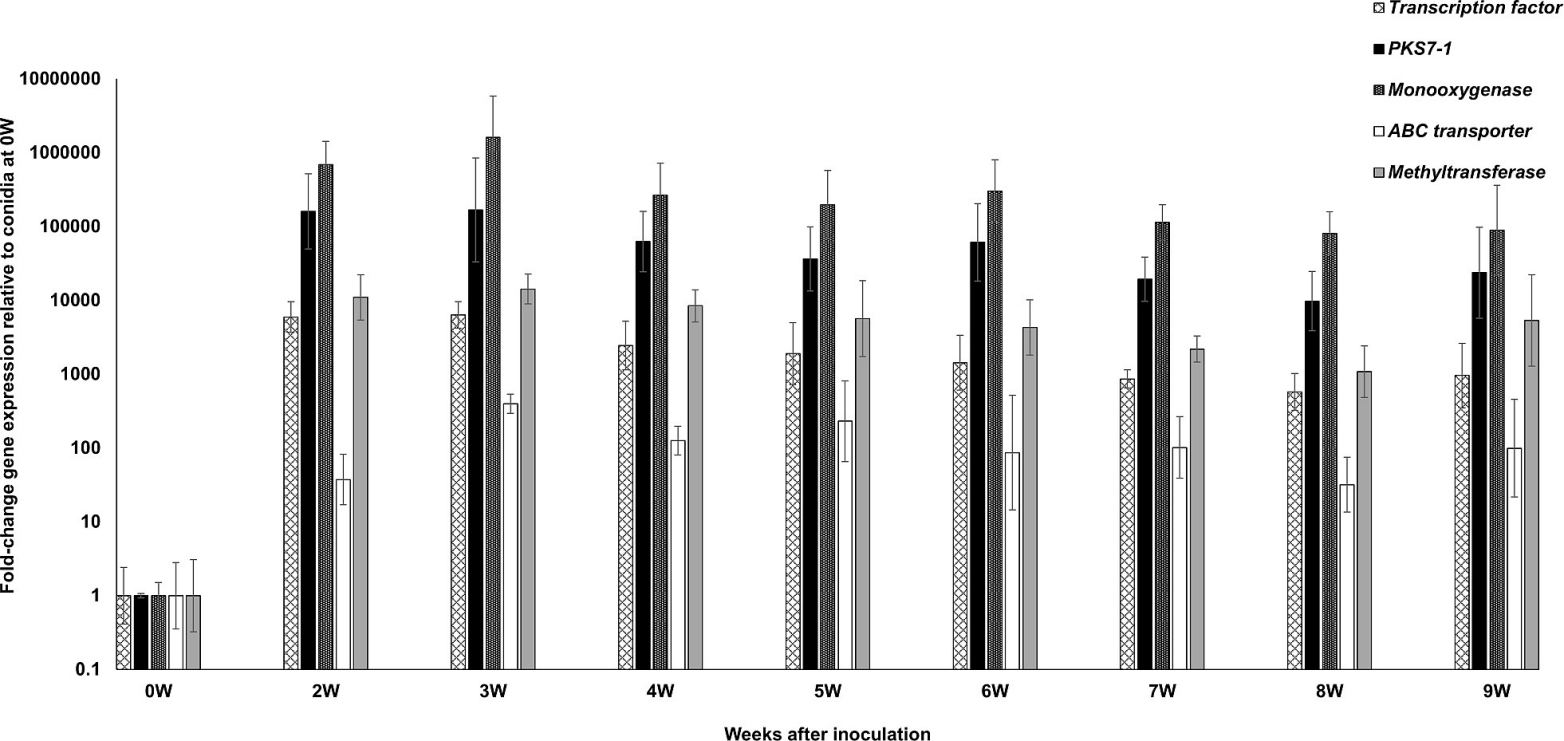
Time course of fold-change gene expression of *PKS7-1* cluster genes in wild type *P*. *fijiensis* isolate 10CR1-24 in infected banana relative to expression in conidia used for inoculation. *PKS7-1* cluster genes assessed include the Zn(II)2 CYS6 transcription factor, *PKS7-1*, the monooxygenase/hydrolase, the O-methyltransferase, and the ABC transporter genes. To determine gene expression, tissue was harvested from infected banana plants at weekly intervals starting at 2 weeks through 9 weeks post-inoculation. Fold-change gene expression is shown relative to the expression at 0 weeks (in conidia) which is set to 1, with error bars indicating standard error from three biological replicates. W = week.

**Fig 2:**
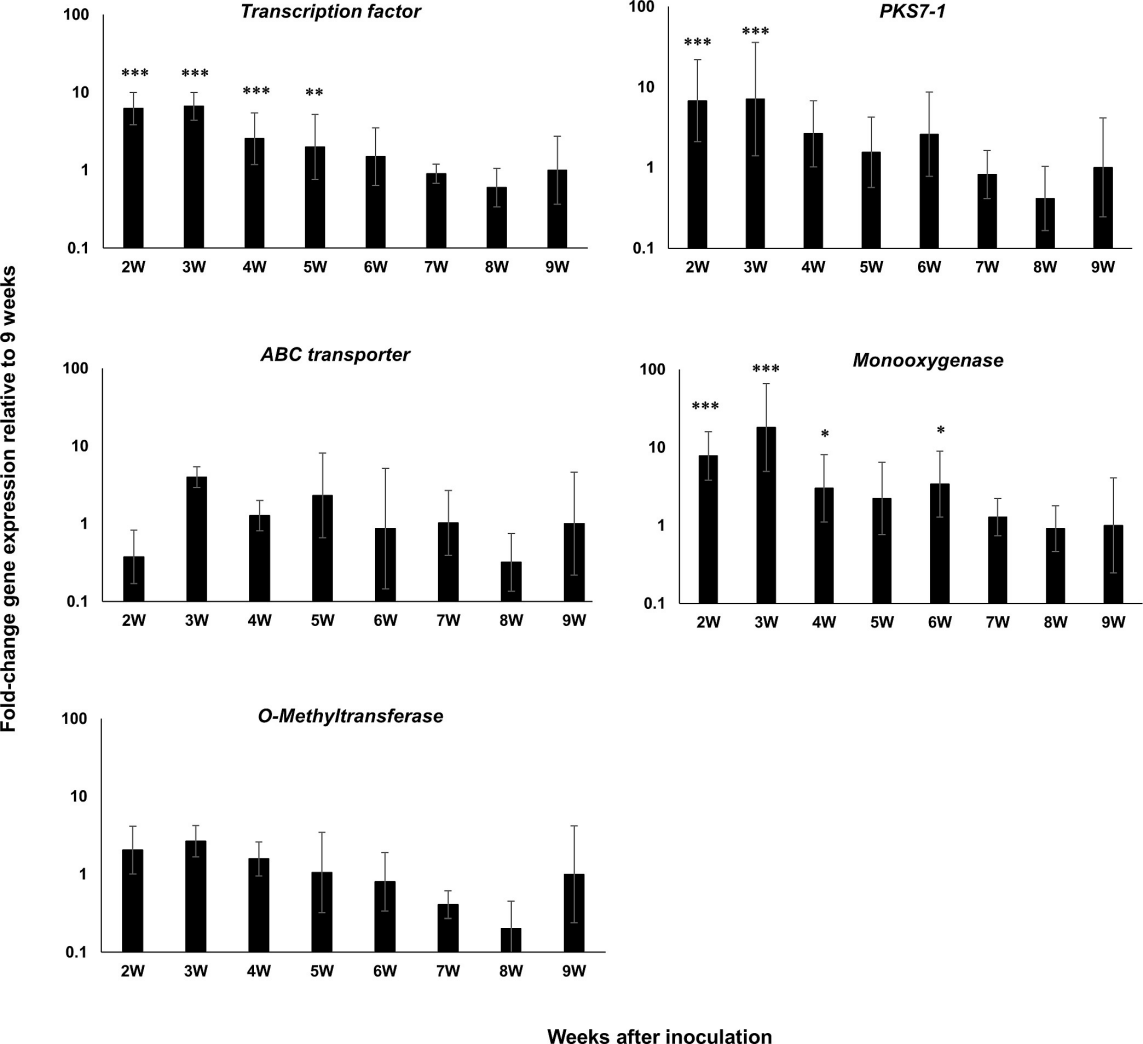
Time course of fold-change gene expression of *PKS7-1* cluster genes in wild type *P*. *fijiensis* isolate 10CR1-24 in infected banana relative to expression at 9 weeks. *PKS7-1* cluster genes assessed include the Zn(II)2 CYS6 transcription factor, *PKS7-1*, the ABC transporter, the monooxygenase/hydrolase, and the O-methyltransferase genes. Tissue was harvested and assayed weekly starting at 2 weeks post-inoculation through 9 weeks. Samples were normalized with reference genes indicated in Table 1. Fold-change gene expression is shown relative to the expression at 9W post-inoculation that is set to 1. Statistical significance was assessed using one-way ANOVA combined with Dunnett’s multiple comparison analysis using the normalized transcript of the 9W sample as the control group. Differences in gene transcript levels considered statistically significant are denoted by asterisks (*P< 0.05, **P< 0.01, and ***P< 0.001). Error bars indicate standard error from three biological replicates. Each biological replicate was tested with three technical replicates.

Our previous work on the *PKS7-1*, *PKS8-2*, and *PKS10-2* clusters included an RNA-Seq analysis of cluster gene expression in infected leaves at a single time point (6 weeks post-inoculation) as compared to mycelial cultures grown *in vitro* [34]. To better characterize expression during infection and disease development, we conducted a time course study of cluster gene expression relative to expression in the conidia used to infect plants, assaying expression of both the PKS genes as well as the other genes located in each cluster [34]. The *P*. *fijiensis* isolate 10CR1-24 [34] was inoculated onto banana plants grown under greenhouse conditions as previously described [35]. For the zero-week time point, we measured expression in the conidia prior to inoculation onto plants. Leaves were harvested weekly starting at 2 weeks post-inoculation, and gene expression was quantified using RT-qPCR as described in Methods. Lesions first appeared between 4 and 5 weeks post-inoculation, followed by lesion expansion and blighting of the leaf tissue extending through 9 weeks post-inoculation. For the *PKS7-1* cluster, we measured expression of the PKS as well as the cluster genes encoding the Zn(II)2 CYS6 transcription factor, the monooxygenase/hydrolase, the O-methyltransferase, and the ABC transporter found in the cluster. Significant increases in expression of all genes could be detected as early as 2 weeks post-inoculation, and expression remained high through 9 weeks (Fig 1). As expression in plants relative to conidia was uniformly high, we chose to measure *in planta* expression relative to 9 weeks in order to determine variations in the expression pattern during the course of the infection process (Fig 2). For the *PKS7-1* cluster, relative expression of genes encoding the transcription factor, PKS, and monooxygenase was highest during the early time points, prior to symptom development and blighting, whereas expression of the O-methyltransferase and transporter was relatively constant throughout the assay period (Fig 2).

**Fig 3:**
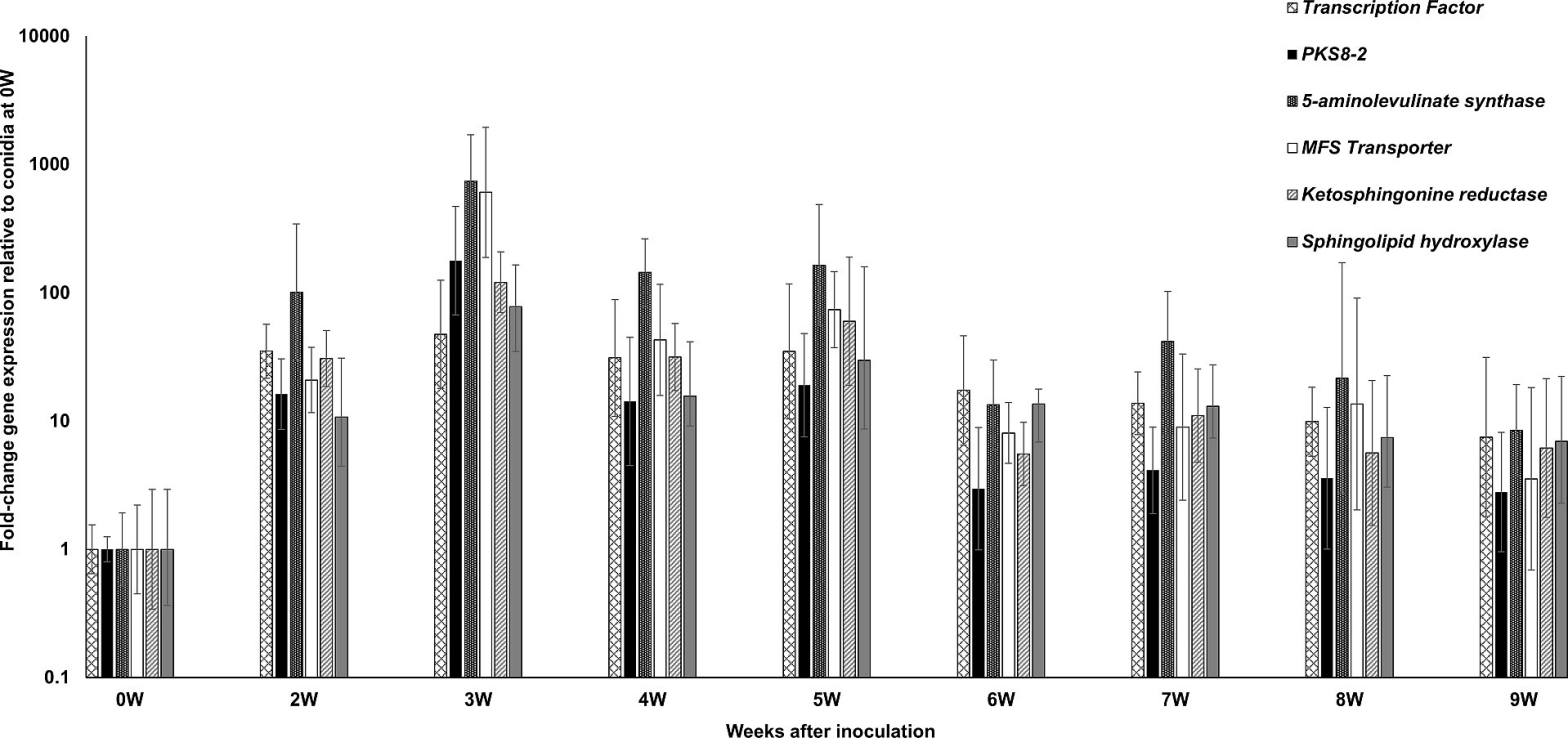
Time course of fold-change gene expression of *PKS8-2* cluster genes in wild type *P*. *fijiensis* isolate 10CR1-24 in infected banana relative to expression in conidia. *PKS8-2* cluster genes assessed include the transcription factor, *PKS8-2*, the 5-aminolevulinate synthase, the MFS transporter, the ketosphinganine reductase, and the sphingolipid hydroxylase genes. To determine gene expression, tissue was harvested from infected banana plants at weekly intervals starting at 2 weeks through 9 weeks post-inoculation. Fold-change gene expression is shown relative to the expression at 0 weeks (in conidia) which is set to 1, with error bars indicating standard error from three biological replicates. W = week.

**Fig 4:**
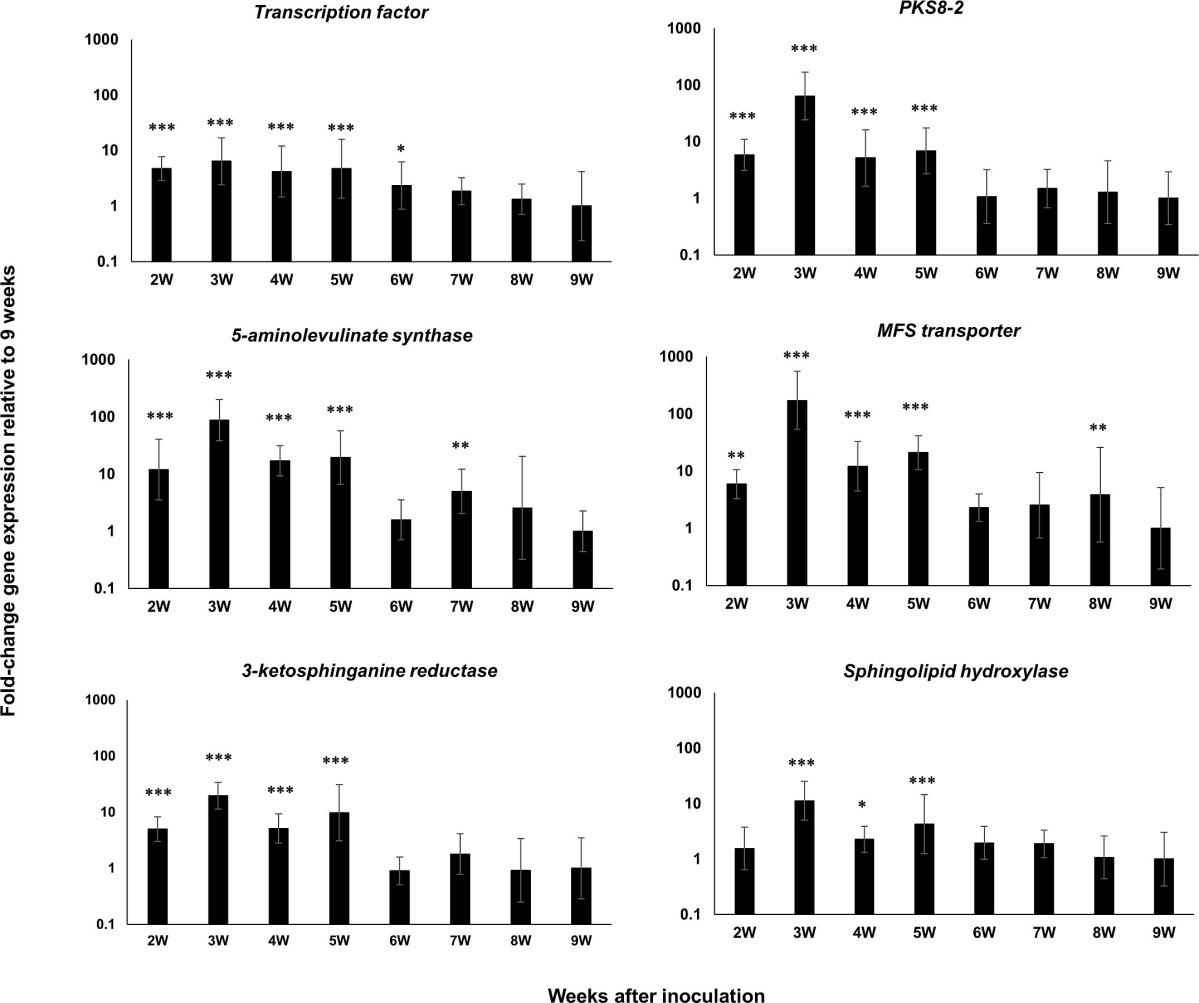
Time course of fold-change gene expression of *PKS8-2* cluster genes in wild type *P*. *fijiensis* isolate 10CR1-24 in infected banana relative to expression at 9 weeks. *PKS8-2* cluster genes assessed include the transcription factor, *PKS8-2*, the 5-aminolevulinate synthase, the MFS transporter, the ketosphinganine reductase, and the sphingolipid hydroxylase genes. Tissue from inoculated banana plants was harvested and assayed weekly starting at 2 weeks post-inoculation through 9 weeks. Samples were normalized with reference genes indicated in Table 1. Fold-change gene expression is shown relative to the expression at 9W post-inoculation that is set to 1. Statistical significance was assessed using one-way ANOVA combined with Dunnett’s multiple comparison analysis using the normalized transcript of the 9W sample as the control group. Differences in gene transcript levels considered statistically significant are denoted by asterisks (*P< 0.05, **P< 0.01, and ***P< 0.001). Error bars indicate standard error from three biological replicates. Each biological replicate was tested with three technical replicates.

For the *PKS8-2* cluster, we measured expression of the PKS as well as the cluster genes encoding the transcription factor, the 5-aminolevulinate synthase, the ketosphinganine reductase, the sphingolipid hydroxylase, and the MFS transporter found in the cluster [34]. As with the *PKS7-1* cluster genes, all genes were strongly upregulated relative to conidia from 2 weeks through 9 weeks post-inoculation (Fig 3). The comparison of cluster gene expression over time relative to 9 weeks (Fig 4) showed that all genes except the gene encoding the sphingolipid hydroxylase were most highly expressed as early as 2 weeks post-inoculation. Expression of the sphingolipid hydroxylase was evident at 3 and 5 weeks post-inoculation. For all genes, high expression extended through the development of visible symptoms at 5 weeks.

**Fig 5:**
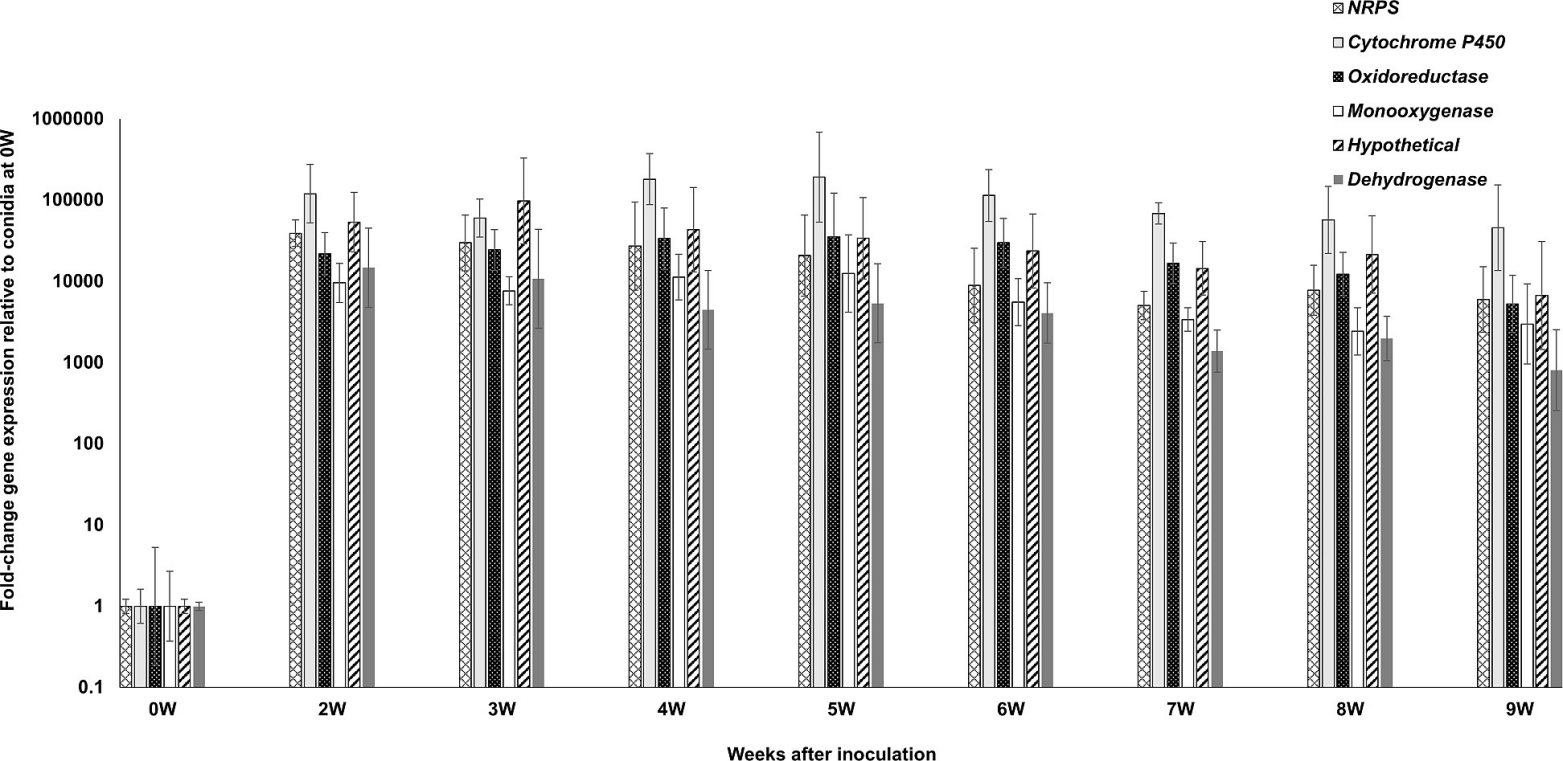
Time course of fold-change gene expression of *PKS10-2* cluster genes in wild type *P*. *fijiensis* isolate 10CR1-24 in infected banana relative to expression in conidia. *PKS10-2* cluster genes assessed include the NRPS, the cytochrome P450, the oxidoreductase, the monooxygenase, a hypothetical gene encoding a protein of unknown function, and the dehydrogenase genes. To determine gene expression, tissue was harvested from infected banana plants at weekly intervals starting at 2 weeks through 9 weeks post-inoculation. Fold-change gene expression is shown relative to the expression at 0 weeks (in conidia) which is set to 1, with error bars indicating standard error from three biological replicates. W = week.

**Fig 6:**
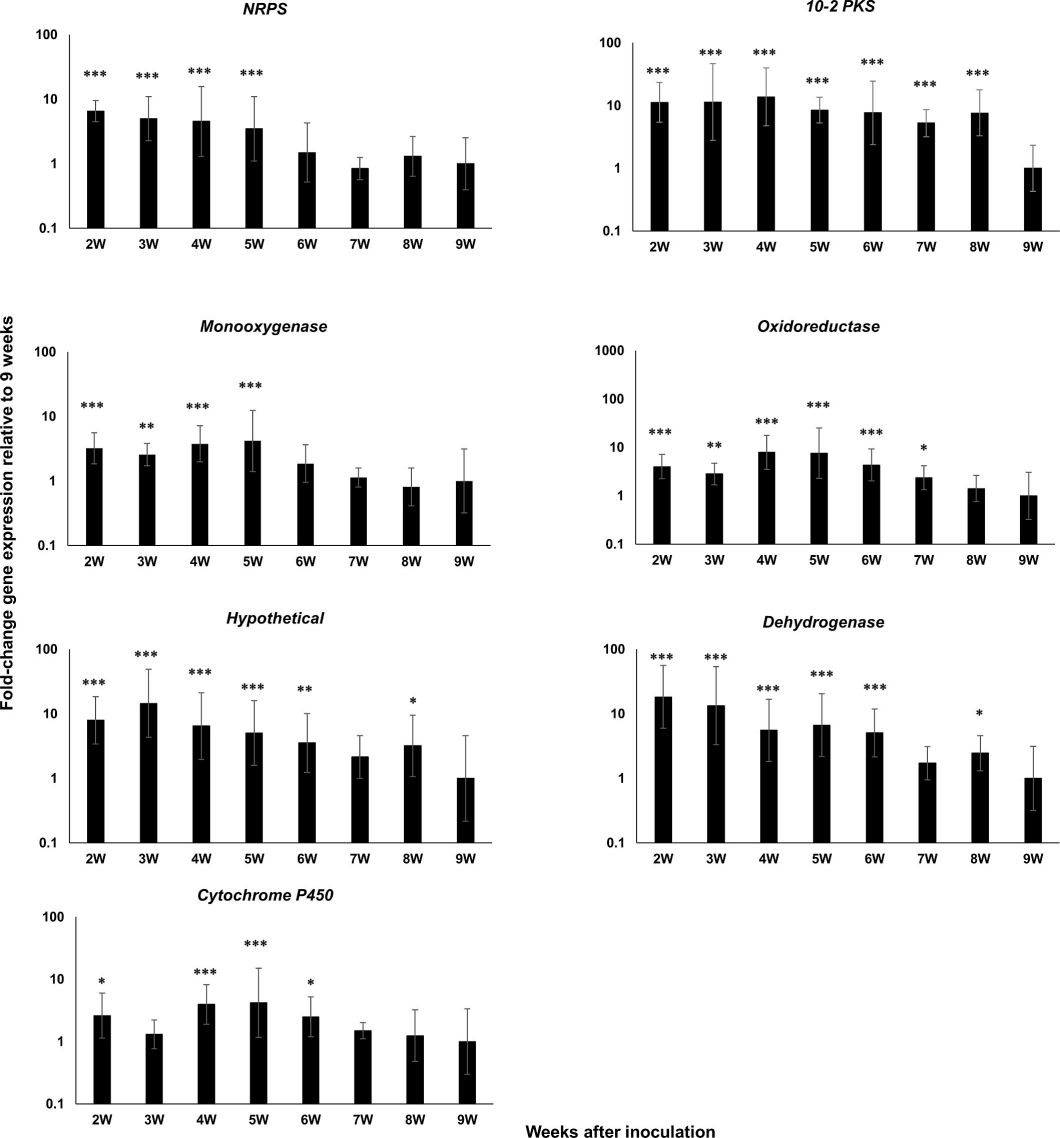
Time course of fold-change gene expression of *PKS10-2* cluster genes in wild type *P*. *fijiensis* isolate 10CR1-24 in infected banana relative to expression at 9 weeks. *PKS10-2* cluster genes assessed include those encoding the NRPS, PKS10-2, the monooxygenase, the oxidoreductase, a protein of unknown function, the dehydrogenase, and the cytochrome P450. Tissue from inoculated banana plants was harvested and assayed weekly starting at 2 weeks post-inoculation through 9 weeks. Samples were normalized with reference genes indicated in Table 1. Fold-change gene expression is shown relative to the expression at 9W post-inoculation that is set to 1. Statistical significance was assessed using one-way ANOVA combined with Dunnett’s multiple comparison analysis using the normalized transcript of the 9W sample as the control group. Differences in gene transcript levels considered statistically significant are denoted by asterisks (*P< 0.05, **P< 0.01, and ***P< 0.001). Error bars indicate standard error from three biological replicates. Each biological replicate was tested with three technical replicates.

For the *PKS10-2* cluster, we measured expression of the *PKS10-2* cluster genes encoding the NRPS, the cytochrome P450, the oxidoreductase, the monooxygenase, and the dehydrogenase as well as a hypothetical protein [34]. As with the other two clusters, all genes were strongly upregulated relative to conidia from 2 weeks through 9 weeks post-inoculation (Fig 5). Comparison of gene expression relative to the 9-week time point showed that *PKS10-2* had significantly greater expression through the 8-week post-inoculation time point, and the remaining genes also showed the highest expression through 5–7 weeks (Fig 6).

### Silencing of *PKS8-2* and *PKS10-2*

**Fig 7:**
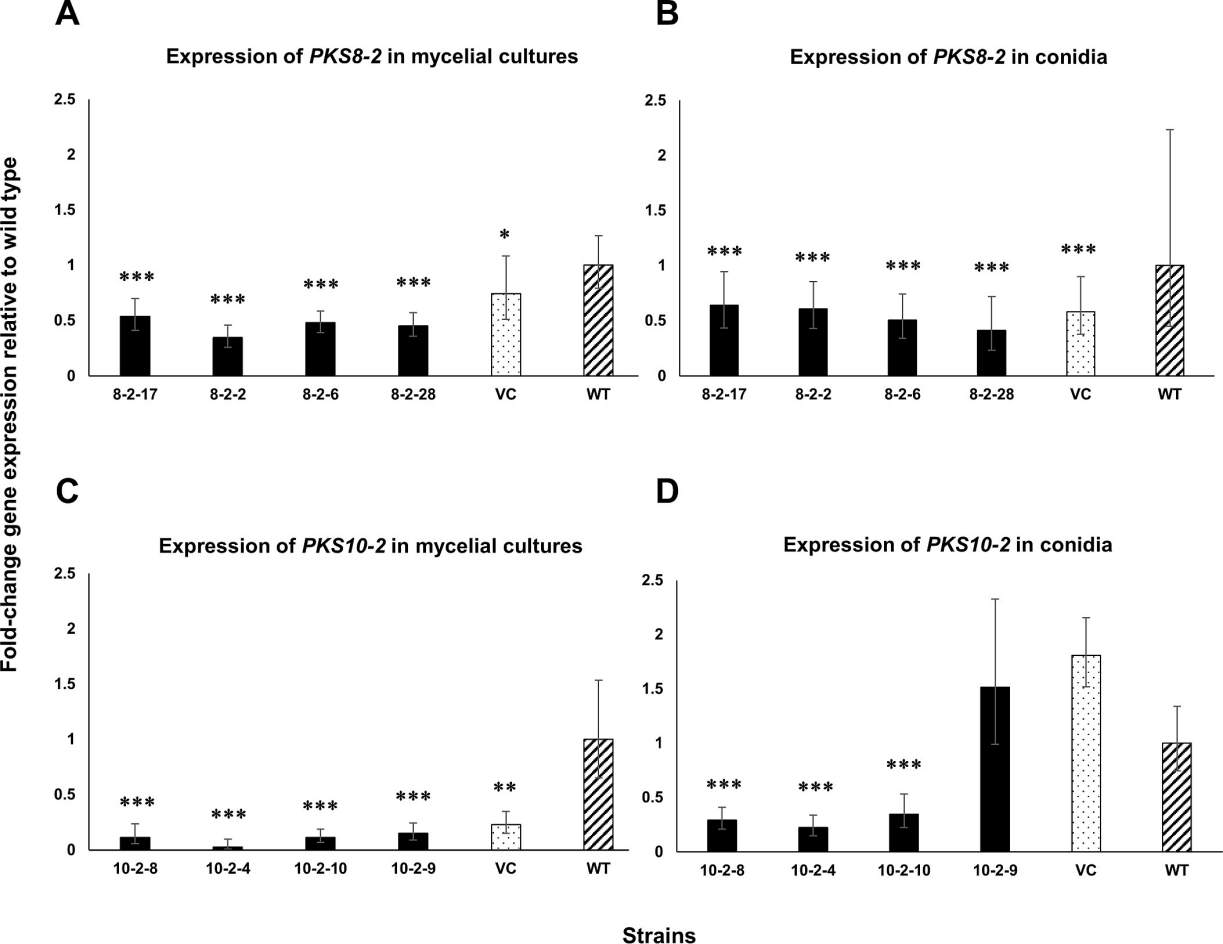
PKS gene expression in silencing transformants, vector control, and wild type. **A)** Expression of *PKS8-2* from mycelial cultures harvested from four-week-old fungal cultures grown on banana leaf—Murashige and Skoog agar. **B)** Expression of *PKS8-2* in conidia harvested from one-week-old mycelial cultures grown on modified V8 medium. **C)** Expression of *PKS10-2* from mycelial cultures harvested from four-week-old fungal cultures grown on banana leaf—Murashige and Skoog agar. **D)** Expression of *PKS10-2* in conidia harvested from one-week-old mycelial cultures grown on modified V8 medium. Controls included the vector control (VC) (dotted bars) and wild type (WT) (hatched bars). Samples were normalized to the actin gene for the *PKS8-2* expression and the ubiquitin gene for the *PKS10-2* expression. Fold-change gene expression in the transformed lines is shown relative to expression in the wild type isolate 10CR1-24. Statistical significance was assessed using one-way ANOVA combined with Dunnett’s multiple comparison analysis using the normalized transcript of 10CR1-24 as the control group. Differences in gene transcript levels considered statistically significant are denoted by asterisks (*P< 0.05, **P< 0.01, and ***P< 0.001). Error bars indicate standard error from three biological replicates for mycelial and conidial expression. Each biological replicate was tested with three technical replicates.

Our previous work documented expression of *PKS7-1*, *PKS8-2*, and *PKS10-2* only in infected plants [34], but we subsequently identified a medium, banana leaf–MS agar (Murashige and Skoog agar with sterile banana leaf pieces), that allowed for *PKS8-2* and *PKS10-2* expression *in vitro*. We thus chose these clusters for further analysis through silencing of the *PKS* genes. RNAi constructs of *PKS8-2* and *PKS10-2* were generated using portions corresponding to the PKS ketosynthase domains, and constructs were transformed into wild type *P*. *fijiensis* isolate 10CR1-24 as described in Methods. Transformants with these constructs were assayed first for construct expression, and then transformants with strong expression of the constructs were screened for silencing using RT-qPCR. Silencing was initially assayed from mycelial cultures grown for four weeks on banana leaf–MS agar, and four transformants for each gene were selected that had significantly lower expression of the PKS genes as compared to the wild type and a vector-transformed control. Transformants were then assayed for expression in conidia used to inoculate plants. Results are shown in Fig 7. For *PKS8-2* (Fig 7A and 7B), expression in the selected silencing transformants differed significantly from both the vector control and wild type when assayed in mycelia (Fig 7A). In conidia, however, expression in these transformants did not differ significantly from the vector control (Fig 7B). For *PKS10-2* (Fig 7C and 7D), all silencing transformants also showed significantly reduced expression in mycelial cultures (Fig 7C). Again, expression in conidia varied from that seen in mycelial culture. Three of the four transformants that showed silencing in mycelial culture (10-2-4, 10-2-8, and 10-2-10) were also strongly silenced for *PKS10-2* in conidia, but one (10-2-9) did not show any silencing relative to either wild type or the vector control (Fig 7D).

### Screening silencing transformants for pathogenicity on banana

Pathogenicity of transformants with the *PKS8-2* and *PKS10-2* silencing constructs was assayed by inoculating banana plants as described in Methods. Briefly, banana plants maintained in *in vitro* culture were transplanted to soil under greenhouse conditions. Plants were inoculated with conidia from wild type 10CR1-24, a vector-transformed control, and the four selected silencing transformants for each gene shown in Fig 7. Disease severity was assayed at 9 weeks post-inoculation by scanning leaves with an Epson Perfection V39 scanner and quantifying the necrotic tissue using Fiji ImageJ package as described in Methods [41, 42].

**Fig 8:**
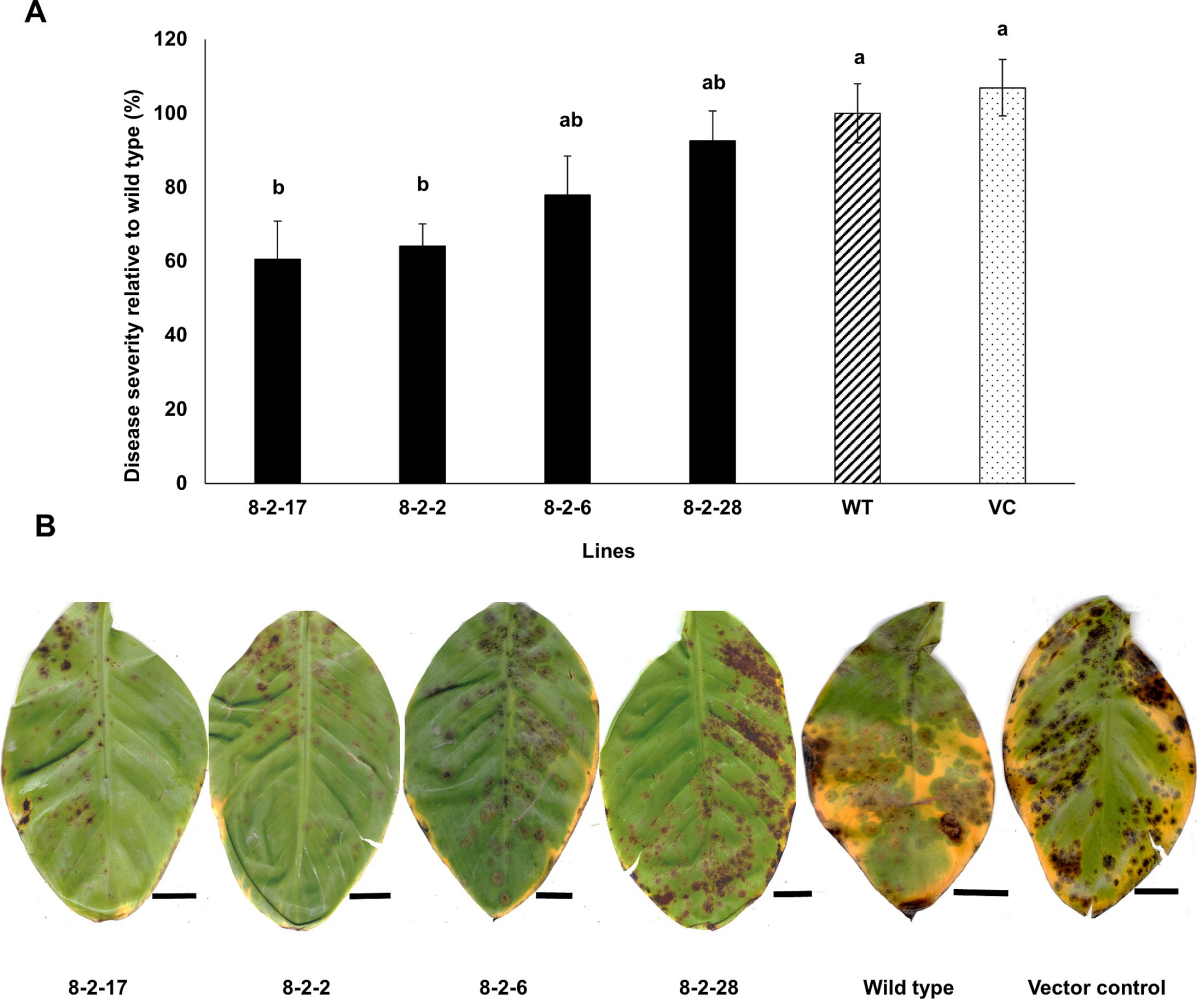
Black Sigatoka disease development in banana inoculated with wild type, vector control, and transformants silenced with the *PKS8-2* silencing construct. **A)** Black Sigatoka disease response as seen in banana plants 9 weeks after inoculation with the wild type (hatched bar), vector control (dotted bar) and silencing transformants (dark bars). Disease severity caused by each isolate was assessed by the Fiji ImageJ program and is shown relative to wild type isolate 10CR1-24. Data shown are results of two independent experiments with 8 plants per strain in each experiment. The within-group differences between the different *PKS8-2* silencing transformants, vector control, and wild type isolate were examined using ANOVA with Bonferroni post hoc test for multiple comparisons. Bars represent Mean ± SE. Different letters on bars indicate statistically significant differences (*p* ≤ 0.05). **B)** Symptoms on banana plants inoculated with *PKS8-2* silencing transformants 8-2-17, 8-2-2, 8-2-6, and 8-2-28 as compared to symptoms on banana inoculated with the vector control and wild type isolate 10CR1-24. Scale bar represents 1 inch.

**Fig 9:**
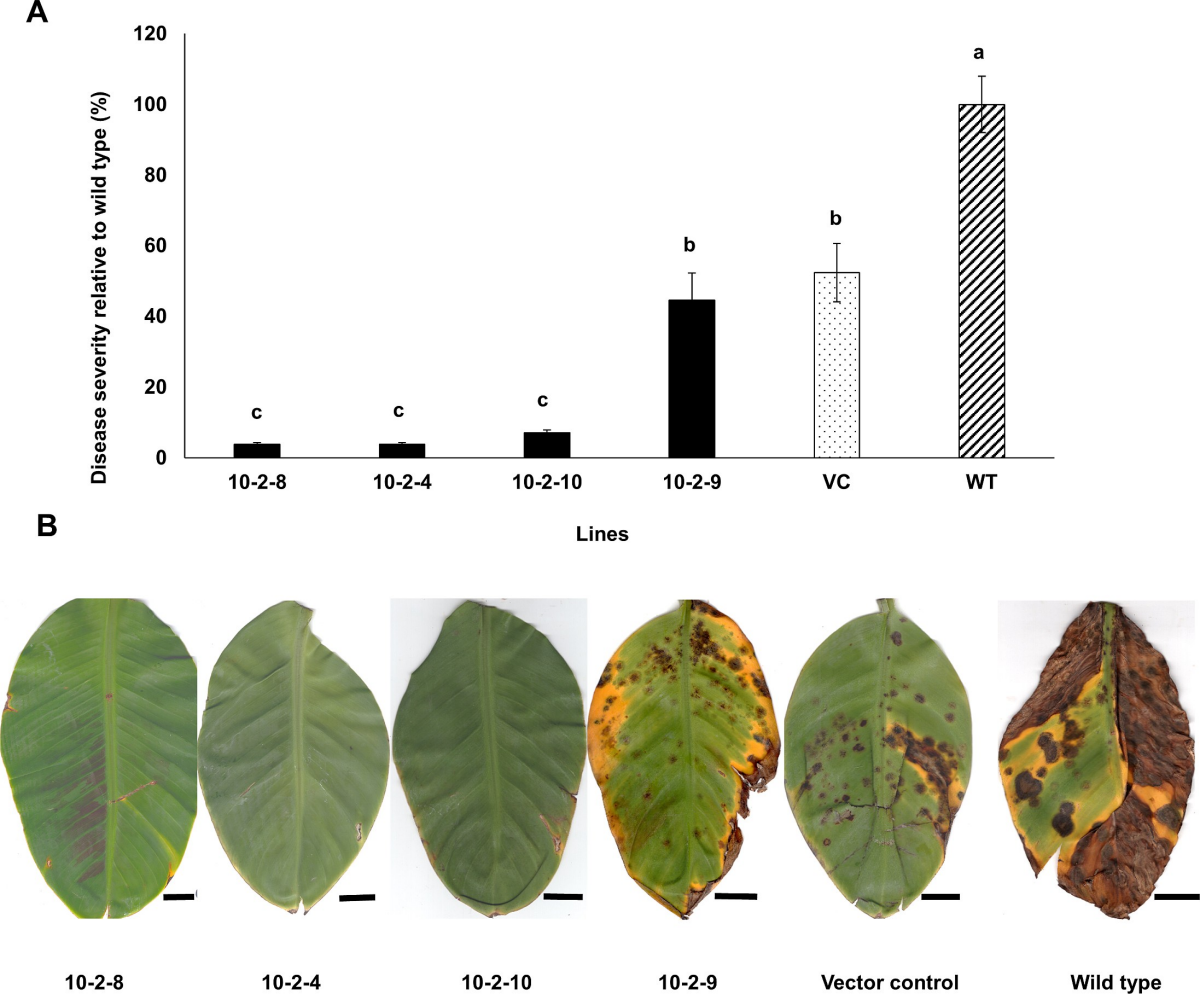
Black Sigatoka disease development in banana inoculated with wild type, vector control, and transformants silenced with the *PKS10-2* silencing construct. **A)** Black Sigatoka disease response as seen in banana plants 9 weeks after inoculation with the wild type (hatched bar), vector control (dotted bar) and silencing transformants (dark bars). Disease severity caused by each isolate was assessed by the Fiji ImageJ program and is shown relative to wild type isolate 10CR1-24. Data shown are results of two independent experiments with 8 plants per strain in each experiment. The within-group differences between the different *PKS10-2* silencing transformants, vector control, and wild type isolate were examined using ANOVA with Bonferroni post hoc test for multiple comparisons. Bars represent Mean ± SE. Different letters on bars indicate statistically significant differences (*p* ≤ 0.05). **B)** Symptoms on banana plants inoculated with *PKS10-2* silencing transformants 10-2-8, 10-2-4, 10-2-10, and 10-2-9 as compared to symptoms on banana inoculated with the vector control and wild type isolate 10CR1-24. Scale bar represents 1 inch.

Data on disease severity are shown in Figs 8 and 9. For the *PKS8-2* silencing transformants there was a significant reduction in disease in plants inoculated with the 8-2-17 and 8-2-2 silencing transformants, but disease caused by two other silencing transformants (8-2-6, 8-2-28) did not differ significantly from wild type or the vector control (Fig 8). For *PKS10-2*, there were significant differences in disease development caused by the *PKS10-2* silencing transformants (Fig 9), and the disease differences correlated with the degree of silencing as shown in Fig 7. Transformants 10-2-4, 10-2-8, and 10-2-10 caused significantly less disease, with symptoms limited to a small number of necrotic lesions with no lesion expansion or blighting of leaf tissue (Fig 9). These were the transformants that showed significant silencing in conidia (Fig 7D). Transformant 10-2-9, which did not show a reduction in *PKS10-2* expression in conidia (Fig 7), was not significantly different from the vector control in symptom development (Fig 9). These data strongly support a role for *PKS10-2* in disease development.

### Bioinformatics analysis of PKS10-2

**Fig 10:**
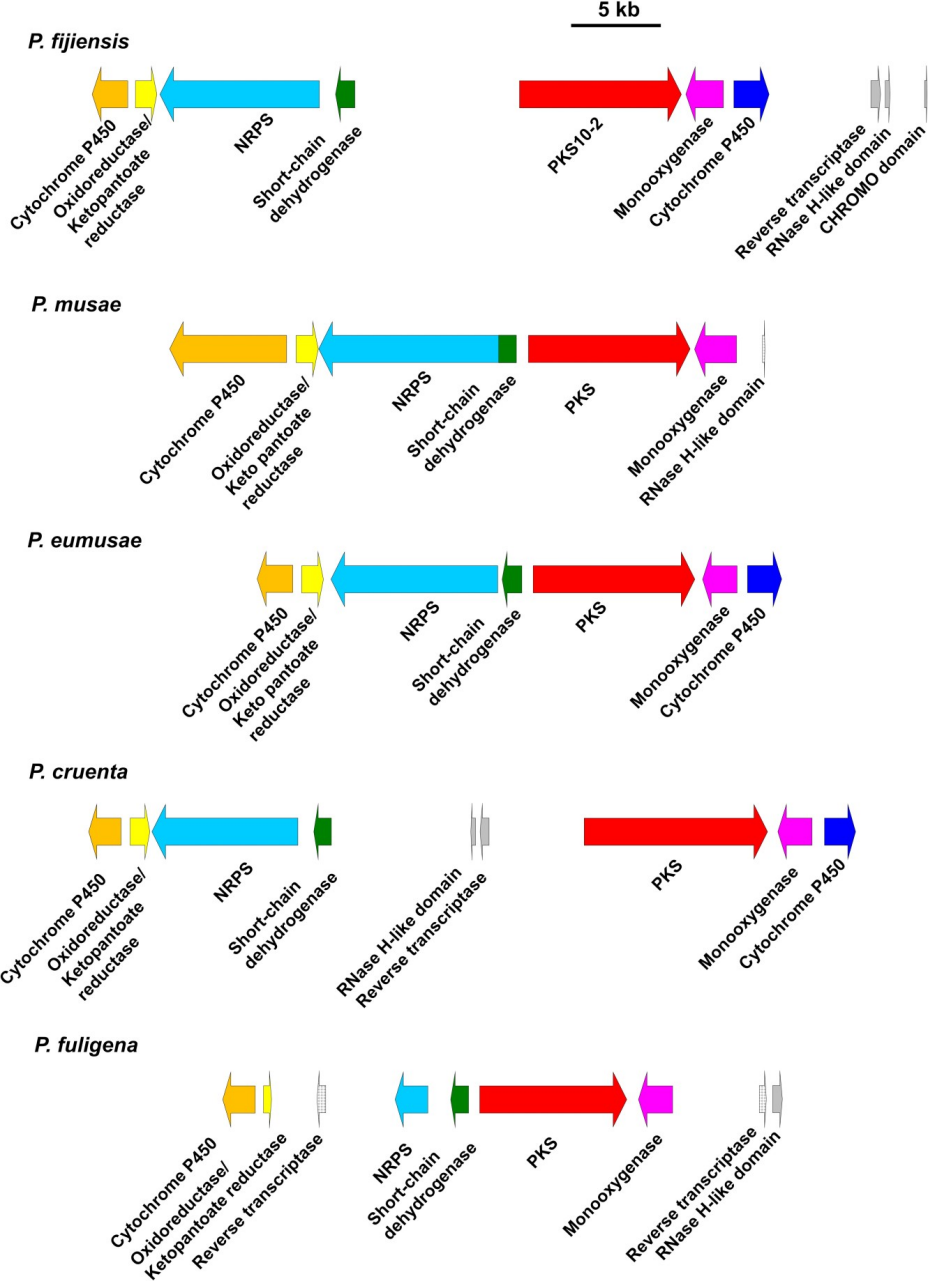
Comparison of *PKS10-2* gene clusters from *Pseudocercospora* species. Putative biosynthetic clusters from *P*. *fijiensis*, *P*. *musae*, *P*. *eumusae*, *P*. *cruenta*, and *P*. *fuligena* are shown, with arrows indicating directionality for each gene. Putative orthologous genes are shown in the same color. Additional conserved domains identified in S5 Table are shown in gray arrows for specific hits and checkered arrows for non-specific hits. Scale bar represents 5 kb.

In previous work [34] we found that the *P*. *fijiensis* PKS10-2 biosynthetic cluster has orthologous sequences from the closely related banana pathogens *P*. *musae* and *P*. *eumusae*. As numerous species in the Mycosphaerellales have been sequenced since then, tblastn searches of the *P*. *fijiensis* PKS10-2 protein sequence were conducted with these newly sequenced genomes. This search identified hits with up to 95% and 98% sequence similarity in the genomes of *Pseudocercospora fuligena* and *Pseudocercospora cruenta*, respectively, suggesting that these species also have orthologous PKS genes (S1 Table). Further blast analyses identified putative orthologs in the *P*. *fuligena*, *P*. *cruenta*, *P*. *musae*, and *P*. *eumusae* genomes of the other genes in the *PKS10-2* biosynthetic cluster (S2 Table). Based on these analyses and the previous analysis that included *P*. *musae* and *P*. *eumusae* [34], the putative *P*. *fijiensis*, *P*. *musae*, *P*. *eumusae*, *P*. *cruenta*, and *P*. *fuligena* gene clusters all encode a cytochrome P450, an oxidoreductase, an NRPS, a short-chain dehydrogenase, a polyketide synthase, and a monooxygenase (Fig 10). A gene encoding a second cytochrome P450 is present in the *P*. *fijiensis*, *P*. *eumusae*, and *P*. *cruenta* gene clusters, but it was not identified from the *P*. *fuligena* genome (Fig 10). The *P*. *musae* genome contains a putative ortholog of the cytochrome P450 gene (S2 Table). However, this gene resides on a different genome assembly scaffold than the rest of the gene cluster. As the *P*. *musae* gene cluster is located at the end of a scaffold, and this cytochrome P450 gene is at the end of another scaffold (S5 Table), more complete genome assembly data would be needed to confirm if these are part of the same gene cluster. Additionally, the predicted *P*. *fuligena* NRPS gene was predicted to be much shorter (approximately 1.8 kb) than the NRPS genes from *P*. *fijiensis* (8.1 kb), *P*. *musae* (10 kb), *P*. *eumusae* (8.5 kb), and *P*. *cruenta* (8.1 kb) (S3 Table). Based on domain analysis using the PKS/NRPS Analysis Web-site [43], the *P*. *fuligena* NRPS has been truncated to contain only an adenylation domain (S4 Table), whereas a functional NRPS must contain, at minimum, a condensation domain, an adenylation domain, and a thiolation or peptidyl carrier protein domain [44]. NRPS sequences from *P*. *fijiensis*, *P*. *musae*, *P*. *eumusae*, and *P*. *cruenta* all contained the necessary domains (S4 Table). Amino acid residues in the predicted adenylation domain binding pockets have been conserved in all these species (S4 Table). The residues in these binding pockets are involved in amino acid substrate recognition [43], however the residues in these adenylation domains are predicted to constitute novel A-domain signatures (S4 Table), thus it is not possible to predict the substrates.

To determine if any additional genes are present in the *Pseudocercospora* gene clusters that were not identified by homology to *P*. *fijiensis* genes, the Conserved Domain Database [45] was used to identify conserved domains in regions with large putative intergenic regions between predicted genes. This analysis identified retrotransposon-like sequences, with conserved domains with homology to those encoding reverse transcriptase and ribonuclease H (Fig 10, S5 Table).

## Discussion

Black Sigatoka causes major disease losses to banana production world-wide, and little is known about the mechanisms the fungus uses to infect and colonize banana tissue. We have been conducting research on polyketide pathways in *P*. *fijiensis*, given our interest in the polyketide toxin cercosporin in the related *Cercospora* species, as well as the importance of polyketides in pathogenicity by diverse fungi. In past work we identified one gene cluster (*PKS8-4*) as being involved in spermagonia production during the sexual reproductive cycle [36]. Another PKS gene cluster (*PKS8-1*) was shown to be conserved across three other *Pseudocercospora* species (*P*. *musae*, *P*. *eumusae*, and *P*. *pini-densiflorae*) and to be strongly up-regulated during disease development [35]. We were unable to definitively identify a role for the *PKS8-1* cluster in disease, however, as *PKS8-1* over-expression mutants did not increase expression in banana over normal expression levels. Here in this study we have focused on three additional polyketide synthase clusters (*PKS7-1*, *PKS8-2*, and *PKS10-2*). Our previous phylogenetic analysis of PKS enzymes showed that PKS8-2 formed a clade with the PKS involved in fumonisin production in *Fusarium verticillioides*, and that PKS10-2 formed a clade with the PKS involved in solanapyrone synthesis in *Alternaria solani*. Fumonisin has been shown to disrupt sphingolipid metabolism [46], and solanapyrone is a known inhibitor of DNA synthesis [47]. Genes clustered with the *PKS* genes in the *P*. *fijiensis* clusters differed significantly from those in the fumonisin and solanapyrone clusters, however, suggesting involvement of different tailoring enzymes and thus different metabolic products. Phylogenetic analysis has not identified any known homologs to PKS7-1.

As an initial test to determine a possible role for the *PKS7-1*, *PKS8-2*, and *PKS10-2* clusters in disease, we conducted a time course analysis of the expression of the PKSs and clustered genes during disease development in banana. Genes assayed in all three clusters were highly expressed in inoculated plants, beginning as early as 2 weeks post-inoculation through the beginning of symptom development (4–5 weeks post-inoculation) and through full symptom development at 9 weeks. Such high expression of all three gene clusters, from the earliest stages of infection through lesion formation, expansion, and tissue blighting, strongly supports an important role for each of these clusters in the ability of *P*. *fijiensis* to colonize and cause disease in banana.

To confirm a role for the PKS gene clusters in disease we successfully developed constructs for silencing of *PKS8-2* and *PKS10-2*. Transformants were selected based initially on expression of the construct, and then were screened for silencing both by mycelial cultures and by conidia. Transformants with the *PKS10-2* silencing construct were identified that were strongly silenced both when grown as mycelium and in conidia. For *PKS8-2*, however, transformants were identified that were strongly silenced when grown as mycelium, but did not show silencing in conidia. The reason for this difference is not known. The strategy used for silencing was the same for both genes and was based on *Agrobacterium*-mediated transformation of hairpin cassettes corresponding to sequences encoding the ketosynthase domains of PKS8-2 and PKS10-2.

Inoculation of banana with silencing strains documented a critical role for the *PKS10-2* cluster in disease development in banana. The silencing transformants shown to be silenced for *PKS10-2* expression both in mycelial cultures and in conidia (10-2-8, 10-2-4, 10-2-10) developed only a few isolated lesions, with no lesion expansion and blighting. Notably, the one silencing transformant that showed *PKS10-2* silencing in mycelial culture but not in conidia (10-2-9) produced disease symptoms comparable to that generated by the vector control. The correlation between the degree of silencing and the dramatic reduction of disease symptoms strongly supports the hypothesis that *PKS10-2* plays an essential role in black Sigatoka disease, and is a promising target for disease control through host-induced gene silencing.

Results were less clear with silencing of *PKS8-2*. Two of the silencing transformants (8-2-17, 8-2-2) were significantly less pathogenic than either the vector control or wild type, but two others (8-2-6, 8-2-28) were not significantly less pathogenic. There was no correlation between the amount of silencing seen in mycelium or conidia with symptom differences in infected plants. Also, symptoms caused by the *PKS8-2* silencing transformants, even those with significantly reduced symptoms, were more severe than seen with the *PKS10-2* silencing transformants. Unlike symptoms caused by the *PKS10-2* silencing transformants where few lesions developed, the *PKS8-2* silencing transformants produced large numbers of lesions, with symptom differences related more to the lack of lesion expansion and blighting.

RNAi silencing is largely conserved throughout the fungal kingdom, but some species have been documented to not have all necessary machinery, suggesting that there may be variability in efficiency between species and strains [48]. Problems with RNAi silencing in *P*. *fijiensis* seem unlikely, however, as Onyilo and co-workers were successful in using RNAi coupled with *Agrobacterium*-mediated transformation to study the role of the *PfHog1* gene in regulating osmotic stress [16]. Further, we were highly successful with targeting *PKS10-2*. Work in *Fusarium graminearum*, using RNAi approaches to target a transcription factor involved in production of the deoxynivalenol mycotoxin, demonstrated that the length of the sequences used for the RNAi construct significantly affects silencing [49]. Although our RNAi constructs for *PKS8-2* and *PKS10-2* both targeted sequences encoding the ketosynthase domain, the sequences were slightly different sequence lengths (799 bp and 436 bp, respectively), so it may be that this difference in length impacted the silencing efficiency. Our inability to see significant symptom suppression with the *PKS8-2* silencing constructs may be due to differences in silencing efficiency or may indicate a less essential role for the *PKS8-2* cluster in *P*. *fijiensis* disease development on banana.

The metabolic product of the *PKS10-2* cluster is not known at this time. However, this cluster has been largely conserved across the fungi *P*. *fijiensis*, *P*. *musae*, *P*. *eumusae*, *P*. *cruenta*, and *P*. *fuligena*, which is consistent with the hypothesis that the cluster may be important for pathogenicity on banana and on other species as well. Specifically, the clusters in all of these species share a cytochrome P450, an oxidoreductase, an NRPS, a short-chain dehydrogenase, a polyketide synthase, and a monooxygenase (Fig 10). A second cytochrome P450 was identified from the clusters of three of the species (Fig 10). No homolog was identified from *P*. *fuligena* (Fig 10), and it is not clear if the *P*. *musae* cluster may contain a second P450. A homolog was identified from *P*. *musae*, but it is located on a different scaffold (S2 Table). As the *P*. *musae* gene cluster and this cytochrome P450 gene are located at the ends of two different scaffolds (S5 Table), further research would be needed to determine whether these two scaffolds are adjacent on the same chromosome. It is notable that the NRPS of *P*. *fuligena* is truncated such that it no longer contains all of the domains considered necessary for function (S4 Table) [44]. The significance of the loss of NRPS domains in *P*. *fuligena* is not yet clear. It may indicate that this cluster is not necessary for pathogenicity in *P*. *fuligena*, or this loss of NRPS sequence may indicate that a different metabolite is produced by this species, since the PKS sequence is still present.

Phylogenetic analyses of the Sigatoka complex pathogens *P*. *fijiensis*, *P*. *musae*, and *P*. *eumusae* have indicated that *P*. *fijiensis* diverged first, and then *P*. *musae* and *P*. *eumusae* form a clade with each other [9, 50]. Another analysis indicates that *P*. *cruenta* and *P*. *fuligena* are most closely related to each other, and then to *P*. *fijiensis* [51]. These three species are more closely related to each other than to *P*. *musae*; *P*. *eumusae* was not included in that analysis [51]. Comparing these species phylogenies to the changes in the *PKS10-2* gene clusters, the closely related species *P*. *musae* and *P*. *eumusae* have similarly sized intergenic regions within the cluster, with minimal space between the PKS and short-chain-dehydrogenase genes (Fig 10). The clusters of those two species differ in that *P*. *musae* has one cytochrome P450 gene with a longer predicted sequence that includes a DUF3328 domain. Interestingly, the portion of this gene corresponding to the DUF3328 domain is homologous to a hypothetical gene near the PKS10-2 gene cluster of *P*. *fijiensis* (S2 Table). Although *P*. *cruenta* and *P*. *fuligena* are most closely related to each other [51], the *P*. *fuligena* cluster is different from all the other species analyzed, since the NRPS gene is truncated and the second cytochrome P450 gene was not detected (Fig 10). The *P*. *fijiensis PKS10-2* gene cluster is most similar to that of *P*. *cruenta*: all of the same cluster genes were detected from both species, in the same gene order and orientation, and there is a larger intergenic region for these species between the genes encoding a short-chain dehydrogenase and the PKS (Fig 10). Since *P*. *cruenta* and *P*. *fuligena* form a sister clade to *P*. *fijiensis* [51], the similarity between the *P*. *fijiensis* and *P*. *cruenta* gene clusters would be expected.

In summary, we have documented high expression of three *PKS* gene clusters (*PKS7-1*, *PKS8-2*, and *PKS10-2*) during symptom development of black Sigatoka disease in banana, supporting a possible role for these pathways in colonization and disease development by *P*. *fijiensis*. Further, our data strongly show that the *PKS10-2* cluster is critical for colonization and symptom development in banana, as transformants silenced for *PKS10-2* are significantly restricted in the ability to form lesions and in lesion expansion and blighting. This PKS cluster is a promising target for control of black Sigatoka through strategies such as host-induced gene silencing.

## Methods

### Time course of expression of PKS gene clusters during disease development

Banana plants grown under greenhouse conditions were inoculated with *P*. *fijiensis* isolate 10CR1-24 as described previously [34]. Total RNA was isolated from infected banana plants starting from 2 weeks post-inoculation using the Spectrum Plant Total RNA kit (Sigma-Aldrich, MO) and was reverse transcribed with the iScript Select cDNA synthesis kit (Bio-Rad) as per the manufacturer’s specifications. For each of the PKS clusters assayed in this study, the cDNA was preamplified with SsoAdvanced PreAmp Supermix (Bio-Rad, CA) using PKS gene cluster-specific primers (Table 1) so as to obtain an unbiased target-specific preamplification of the limited amounts of fungal nucleic acid transcripts. The primers for the *PKS7-1* cluster preamplification included genes for the Zn(II)2 CYS6 transcription factor, PKS7-1, the monooxygenase/hydrolase, the O-methyltransferase, and ABC transporter genes along with the reference genes (Table 1). The primers for the *PKS8-2* cluster assay included genes for the transcription factor, PKS8-2, the 5-aminolevulinate synthase, the ketosphinganine reductase, the sphingolipid hydroxylase, and MFS transporter genes in addition to the reference genes. The primers for the *PKS10-2* cluster preamplification included genes for the NRPS, PKS10-2, cytochrome P450, oxidoreductase, monooxygenase, dehydrogenase, and hypothetical genes along with the reference genes. The thermal cycling protocol included an initial denaturation step of 95°C for 3 minutes, followed by 12 cycles of 95°C for 15 seconds and an annealing step of 58°C for 4 minutes. The preamplified cDNA was diluted to a ratio of 1:5 with Low EDTA TE buffer (USB Corp., OH) and used as templates in qPCR assays that were set up with iQ SYBR Green Supermix (Bio-Rad). The thermal cycling parameter included an initial denaturation step of 95°C for 2 minutes, followed by 45 cycles of 95°C for 10 seconds, target-dependent annealing temperature for 30 seconds, and 72°C for 30 seconds in conjunction with a plate read. Melt curves were used to confirm the amplification of a single product for each reaction. Transcript levels of the target genes were normalized using two reference genes that amplified with the same efficiency as that of the target gene. Fold-change gene expression was determined by the 2^−ΔΔCT^ method by comparing the normalized transcript levels at each week to that of the normalized transcript levels of either the conidia or that of the 9 weeks post-inoculation levels. Expression of genes was statistically analyzed by one-way ANOVA combined with Dunnett’s Multiple Comparison post hoc analysis using the expression at 9 weeks post-inoculation as the control group. Statistical analyses were performed using SPSS 27.0 statistical software (IBM, IL).

**Table 1:** Primer sequences and reference genes used for expression assays.

Cluster	Gene	Primer sequence	Reference genes	Amplicon Size (bp)
*PKS7-1*	*PKS7-1*	ATCGGTTTGATGCACAATCACATCT	*Actin*	293
		TGGTATGAAATAGGTCCCGACTTGCT	*β-tubulin*	
	*Methyltransferase*	AGTGCACGGCTGAGCAATGTC	*Tfc1*	289
		GGCTTCGCTCTCGAGCATTATGT	*Sac7*	
	*Transcription factor*	CAGCCTGCGACTTCTGCCATACT	*Actin*	83
		GGCCATGTTAAGCGAGCTGC	*β-tubulin*	
	*ABC transporter*	CTCTTGTAGGTCCCTCTGGGTCTG	*Tfc1*	302
		TGTGTTTGCGATGCCTTGGA	*Sac7*	
	*Monooxygenase*	TATGGCAAAACGCTTGTCGGC	*Actin*	111
		AACACCGTCAGCTCCCACGA	*β-tubulin*	
*PKS8-2*	*PKS8-2*	TCATGTCGCGTTCTGCCGGT	*UBC6*	200
		GGCGAAGTTCTGGTCTCTCAGGA	*Actin*	
	*PKS8-2* ^a^	GGACAAAGGCGATGCTCGCT	*UBC6*	136
		GCGGACATTGGCATTTTCGT	*Actin*	
	*Transcription factor*	TTGGTGCAGGCAATCATCGC	*β-tubulin UBC6*	208
		AGCTTCTGTTCCACGCGCTG		
	*5-Aminolevulinate synthase*	CATTCGAGCCGGTTATGGTCTG	*Actin*	174
		GGTTTCGTCTCCCAGTTTTCCAT	*β-tubulin*	
	*3-Ketosphinganine reductase*	AACAACTATCTGACAGCCGCATTCG	*UBC6*	175
		CATCGTACCCAGGCAGTCCAA	*Sac7*	
	*MFS transporter*	AAAGCAGCCGTGCGTTTGCG	*Actin*	262
		ATCGAGCCGAGCAGGAAGAGCA	*β-tubulin*	
	*Sphingolipid hydroxylase*	TGCGTTGATGGAATGGGTGG	*Tfc1*	171
		GATATCAGCGCCGGTCAACTTC	*Sac7*	
	*PKS8-2 construct*	GATAACTTGTGCGTTTGTCAAGC	*β-tubulin*	350
		GGTCCTTGGCGAACATCTC	*UBC6*	
*PKS10-2*	*PKS10-2*	TGCCATTGGGCGGGTTTTCG	*Pda1*	248
		GGCCTGTGACGTTCCATGGAGT	*β-tubulin*	
	*PKS10-2* ^b^	GCTTCGAATCCGCAGGCATT	*UBC6*	319
		TCGAGGCCGAGAACGAGGTT	*β-tubulin*	
	*Oxidoreductase*	GCACGATATGGGATGGAACGTC	*Tfc1*	201
		CACCAGTTGAACCAGCGTTCG	*Sac7*	
	*Cytochrome P450*	CAGTGTCCTGGTGGCCTGTCTCTCT	*UBC6*	327
		CCACGTTCGAAAGGCCAGGT	*Sac7*	
	*NRPS*	GCTTCAATCCCCAAAGGAGTCG	*UBC6*	331
		TCTGCGTTCCATTCTGGAGTGGT	*β-tubulin*	
	*Monooxygenase*	CCCAACTTGACCGACGCAAT	*UBC6*	165
		TGCCCGCAAGATACAGAAAG	*Sac7*	
	*Dehydrogenase*	CTGTTGCCCATGCTGTGCCA	*UBC6*	320
		AGCAAGAGCTTCGCGATCGC	*Actin*	
	*Hypothetical*	TACCCCGTGGAGATCCAAGTGG	*Actin*	268
		CGAAGATCGGCTGTGGTCCAATT	*β-tubulin*	
	*PKS10-2 construct*	TGATGCCGGAGCTTCTCCCATTT	*Actin*	151
		CGGAGCTGACATCGACACC	*β-tubulin*	
N/A	*Actin*	CTTGACTCCGGTGACGGTGTCACTC	N/A	252
		CGTCAGGAAGCTCGTAGGACTTCTC		
	*β-tubulin*	TGGCACGTCTGATCTCCAGC	N/A	128
		GCGGAAGAGCTGACCGAATGG		
	*UBC6*	GGATGCGGAGAATTGGAAGTGG	N/A	226
		TCAGCAATCTCGCAACCATCAC		
	*Tfc1*	CCAGACTTCCAGCTTCGTAGC	N/A	225
		GTTCTTGGCAGTCACCTGTCC		
	*Pda1*	GAGCAGTCACGTTGACAAGAGC	N/A	265
		CCTGTTTGGTGATTGCGTGC		
	*Sac7*	GCGCCCGAGATCAAATCTGTCA	N/A	232
		CCATCGCATCCTGGATTGGC		

^a^ primers used for assaying *PKS8-2* expression in silencing transformants

^b^ primers used for assaying *PKS10-2* expression in silencing transformants

UBC6 = Ubiquitin; Tfc1 = Transcription factor class C 1; Pda1 = Pyruvate dehydrogenase alpha 1; Sac 7 = Suppressor of actin 7

N/A = not applicable

For each gene assayed in RT-qPCR assays from Figs 1–7, the table indicates the gene assayed, the forward and reverse primer sequences, two reference genes for each gene of interest, and amplicon sizes.

### Generation of *Agrobacterium*-compatible RNAi transformation vectors

To generate the RNAi constructs of *PKS8-2* and *PKS10-2*, portions corresponding to the PKS ketosynthase domains were amplified from genomic DNA from *P*. *fijiensis* isolate 10CR1-24 [34]. Primers used were 5’ -GGGGACAAGTTTGTACAAAAAAGCAGGCTTAAGATGCCTCGATGTTTAG- 3’ and 5’ -GGGGACCACTTTGTACAAGAAAGCTGGGTAAGCCGATGTACCTTGTAC- 3’ for *PKS8-2*, and 5’ -GGGGACAAGTTTGTACAAAAAAGCAGGCTTAAAATGGGAGAAGCTCCGGCATCA- 3’ and 5’ -GGGGACCACTTTGTACAAGAAAGCTGGGTACAAATGAATTGATGCTTGTCC- 3’ for *PKS10-2*, which were designed to add attB sites for cloning into the vector pDONR221, using Gateway technology. Gateway technology was further used to transfer the PKS sequences into the RNAi-compatible vector pTROYA [52]. XbaI and EcoRI was used to transfer the PKS RNAi expression cassettes from pTROYA to a modified pEarleyGate 100 with pGPD:GFP and a hygromycin selectable marker [36], which removes the GFP expression cassette in the process (S1 Fig). The resulting plasmids were then transformed into *Agrobacterium tumefaciens* strain EHA105. *P*. *fijiensis* isolate 10CR1-24 was transformed as previously described [36].

### Screening of silencing transformants under *in vitro* conditions

Transformants and the wild type isolate 10CR1-24 were grown on sterile cellophane disks overlaid on banana leaf—MS agar, made by placing sterile banana leaf pieces (*ca* 1 x 1 cm) harvested from plants grown under tissue culture conditions in petri dishes and covering with heat-sterilized Murashige and Skoog medium [53] containing 6g/L Millipore-Sigma agar. The cultures were grown at 28°C under an 18h light/6h dark photoperiod for four weeks. Total RNA was isolated from the mycelium using the Spectrum Plant Total RNA kit (Sigma-Aldrich, MO) and reverse transcribed with iScript Reverse Transcription Supermix for RT-qPCR (Bio-Rad, CA) as per the manufacturer’s specifications. Gene expression was quantified using qPCR as described above. Primers for detecting the transcript levels in the transformants were designed to anneal in the region of the ketosynthase domain that was not part of the silencing construct so that the transcripts quantified would be from the fungal PKS gene rather than the silencing construct. Transcript levels of the PKS genes were normalized using two reference genes that amplified with the same efficiency as that of the target gene. Fold-change gene expression was determined by using the 2^−ΔΔCT^ method wherein the normalized transcript level of each transformant and the vector control was compared to the normalized transcript levels of wild type isolate.

In order to confirm that silencing was also occurring in the conidia used to inoculate banana plants, we examined the expression of *PKS8-2* and *PKS10-2* genes at the conidial stage. A 500-μl solution of hyphal fragments generated by macerating three-week-old fungal cultures grown on PDA was plated on modified V8 medium (0.2g/L CaCO_3_, 100 ml/L of V8 juice and 20g/L Difco agar (Becton- Dickenson, NJ) and incubated at 18°C under continuous cool-white fluorescent and black light for one week. The conidia were harvested by scraping the plates with 3 ml of water using a cell spreader, and collecting the spore suspension with a pipet. After removing the water by filtering through sterile Miracloth (Millipore-Sigma, MO), total RNA was isolated and reverse transcribed as described above. Fold-change gene expression was determined by comparing the normalized transcript level of the transformant and vector control to the normalized levels of the wild type using the 2^−ΔΔCT^ method. Gene expression of all the isolates screened under *in vitro* conditions was statistically analyzed by one-way ANOVA with Dunnett’s Multiple Comparison post hoc analysis using the expression of wild type isolate as the control group.

### Banana inoculation

Tissue cultured banana plants belonging to the Grand Nain cultivar (kindly provided by Miguel Muñoz, Dole Food Company) were maintained on modified Murashige and Skoog medium as previously described [34] at 28°C under an 18h light/6h dark photoperiod with cool-white fluorescent light. Banana plants that had rooted under *in vitro* conditions were transferred to potting mix and grown in the North Carolina State University Phytotron facility under a 12h light/12h dark photoperiod at 26°C during the day and 22°C during the night. Conidia were obtained from each of the strains as described above for the conidia gene expression experiments. Both sides of the leaves of banana plants 4 weeks post-planting were sprayed with 25 ml of a 5x10^3^ conidial suspension in 0.5% Tween 20 solution. Plants were covered with clear plastic bags for one week post-inoculation to maintain high humidity conditions, and symptom development was monitored weekly.

### Assessing black Sigatoka disease severity

Disease severity was assayed at 9 weeks post-inoculation by scanning leaves with an Epson Perfection V39 scanner. Image analysis was carried out by using the Fiji ImageJ package [41, 42]. After importing the image into Fiji ImageJ, the scale was determined based on the assignment of known distance in pixels to a known distance in metric unit (cm). Threshold color segmentation by Fiji ImageJ allowed the image pixels to be separated according to the color intensities of necrotic areas compared to the healthy areas of the leaves. First, total leaf area was determined by isolating the leaves from the background and quantified using the appropriate color threshold values for brightness. Then, the necrotic areas were segmented using the appropriate color threshold values for hue. These values were used in determining percentage of leaf area occupied by necrotic lesions and further computing the disease severity of each strain compared to the wild type. For each strain, an average of 33 leaves were scanned and analyzed to determine black Sigatoka disease severity. In each experiment, banana plants inoculated with the silencing transformants, vector control and the wild type isolate were represented by eight biological replicates, and the entire study was repeated once again. To test the role of silencing in symptom development, the disease severity of the plants inoculated with the silencing transformants was compared to the disease severity in banana plants inoculated with wild type and statistically analyzed using ANOVA combined with Bonferroni Multiple Comparison post hoc analysis.

### Comparison of orthologous gene clusters to *P*. *fijiensis PKS10-2* biosynthetic cluster

PKS10-2 was used as a query to perform tblastn against 33 genome sequences within the Mycosphaerellales to identify putative orthologs using BLAST+ (S1 and S6 Tables) [54]. To identify genes in the orthologous gene clusters, protein sequences encoded by the putative *P*. *fijiensis PKS10-2* gene cluster were used for blastp searches against the annotated protein sequences from *P*. *fuligena*, *P*. *musae*, and *P*. *eumusae*. Protein sequences found by this method from *P*. *fuligena*, *P*. *musae*, and *P*. *eumusae* were then used for blastp against the *P*. *fijiensis* protein sequences to verify that the sequences are each other’s best hit. Tblastn searches of *P*. *fijiensis* sequences were also conducted against the *P*. *fuligena*, *P*. *musae*, and *P*. *eumusae* genomes, to identify additional putative homologs from these species. *P*. *fuligena*, *P*. *musae*, and *P*. *eumusae* sequences identified using this method were then used as queries against the *P*. *fijiensis* protein sequences using blastx. Because *P*. *cruenta* does not have an available protein annotation, homologs of *P*. *fijiensis* genes were identified from *P*. *cruenta* using tblastn, with blastx used to verify that the sequences are each other’s best hits (S2 Table).

For each putative cluster gene from *P*. *fuligena* and *P*. *cruenta*, as well as adjacent annotated protein-encoding sequences in *P*. *fuligena*, conserved domains from the Conserved Domain Database [45] were identified using the blastp or blastx algorithms (S3 Table). For genes with corresponding protein annotations from *P*. *fuligena*, InterProScan (https://www.ebi.ac.uk/interpro/) and KOG [55] descriptions were identified (S3 Table). Additionally, blastp or blastx was used for each protein or genomic DNA sequence, respectively, to identify the top ten hits from the non-redundant protein database in NCBI (S3 Table). To predict NRPS domains from the sequences from *P*. *fijiensis*, *P*. *musae*, *P*. *eumusae*, *P*. *fuligena*, and *P*. *cruenta*, the PKS/NRPS Analysis Web-site [43] was used with predicted protein sequences from each NRPS. This analysis provides the predicted domains, their locations within the protein, the amino acids in the putative binding sites of the adenylation domains, and if known, the predicted substrate of the adenylation domain (S4 Table). To identify any additional putative genes, sequences corresponding to sizeable putative intergenic regions between genes in the *P*. *fijiensis*, *P*. *musae*, *P*. *eumusae*, *P*. *cruenta*, and *P*. *fuligena* gene clusters were used as blastx queries in the Conserved Domain Database (S5 Table).

## Supporting information

S1 Fig Cloning strategy to generate *Agrobacterium* mediated transformation-compatible PKS RNAi constructs. To generate the *Agrobacterium* mediated transformation-compatible PKS RNAi constructs, a portion of the PKS gene was amplified by PCR with primers that add Gateway attB sites at the ends of the PCR product. A BP reaction was then performed between this PCR product and pDONR221 (A) to generate an entry clone (B) with the PKS sequence contained in the pDONR221 backbone. An LR reaction was then performed with this entry clone and pTROYA (C) to generate an expression clone (D) with a fungal promoter to drive expression of the PKS sequence in inverted repeats, followed by a fungal terminator, in the pTROYA backbone. The PKS RNAi cassette was then transferred from the pTROYA backbone into a modified pEarleyGate 100 plasmid (E) [36] such that the final plasmid in the pEarleyGate 100 backbone contains both the PKS RNAi expression cassette and a hygromycin resistance cassette (F). Plasmid maps were generated in Geneious 6.1.8 (https://www.geneious.com). The figure shows the *PKS10-2* silencing constructs; *PKS8-2* silencing constructs were generated in the same way.
(TIF)

S1 Table Homologs of PKS10-2 among members of Mycosphaerellales. Tblastn results using the *P*. *fijiensis* PKS10-2 protein sequence (NCBI accession XP_007931278.1) as a query, against 33 genome sequences within the Mycosphaerellales (S6 Table). For each hit, the table indicates the species, the genome assembly scaffold, the percent identity and similarity of the hit, the bitscore, the E-value, the gap open value, the number of mismatches, the length of the hit, the query sequence start and end location, and the subject sequence start and end location.
(XLSX)

S2 Table Results of reciprocal blast searches to identify homologous sequences to those encoded by the *P*. *fijiensis PKS10-2* biosynthetic cluster. For each protein sequence encoded by the *P*. *fijiensis PKS10-2* biosynthetic cluster, blastp and/or tblastn searches were performed to identify homologous sequences encoded by the *P*. *fuligena* and *P*. *cruenta* genomes. The sequence with the best hit from each search was then used for blastp or blastx analysis to verify that the two sequences are each other’s best hit. The table indicates the NCBI accession of the query sequence, a description of the query sequence, the location where the query sequence is encoded in the genome, the NCBI accession or genome assembly scaffold of the subject sequence, the percent identity and similarity of the hit, the bitscore, the E-value, the gap open value, the number of mismatches, the length of the hit, the query sequence start and end location, and the subject sequence start and end location. Sequences represented in Fig 10 are indicated by red text. A) *P*. *fuligena* homologs of *P*. *fijiensis* sequences encoded by the *PKS10-2* gene cluster, identified by blastp analysis; B) *P*. *fijiensis* homologs of *P*. *fuligena* biosynthetic cluster sequences, identified by blastp analysis; C) *P*. *fuligena* homologs of *P*. *fijiensis* sequences encoded by the *PKS10-2* gene cluster, identified by tblastn analysis; D) *P*. *fijiensis* homologs of *P*. *fuligena* biosynthetic cluster sequences, identified by blastx analysis; E) *P*. *cruenta* homologs of *P*. *fijiensis* sequences encoded by the *PKS10-2* gene cluster, identified by tblastn analysis; F) *P*. *fijiensis* homologs of *P*. *cruenta* biosynthetic cluster sequences, identified by blastx analysis; G) *P*. *musae* homologs of *P*. *fijiensis* sequences encoded by the *PKS10-2* gene cluster, identified by blastp analysis; H) *P*. *fijiensis* homologs of *P*. *musae* biosynthetic cluster sequences, identified by blastp analysis; I) *P*. *musae* homologs of *P*. *fijiensis* sequences encoded by the *PKS10-2* gene cluster, identified by tblastn analysis; J) *P*. *fijiensis* homologs of *P*. *musae* biosynthetic cluster sequences, identified by blastx analysis; K) *P*. *eumusae* homologs of *P*. *fijiensis* sequences encoded by the *PKS10-2* gene cluster, identified by blastp analysis; L) *P*. *fijiensis* homologs of *P*. *eumusae* biosynthetic cluster sequences, identified by blastp analysis; M) *P*. *eumusae* homologs of *P*. *fijiensis* sequences encoded by the *PKS10-2* gene cluster, identified by tblastn analysis; N) *P*. *fijiensis* homologs of *P*. *eumusae* biosynthetic cluster sequences, identified by blastx analysis.
(XLSX)

S3 Table Putative orthologous gene clusters to *P*. *fijiensis PKS10-2*, from *P*. *fuligena* and *P*. *cruenta*. For each gene in the putative biosynthetic cluster, the table indicates the description in Fig 10, the accession of the putative *P*. *fijiensis* homolog protein in cases where they are each other’s best blast hit, the scaffold wherein the *P*. *fuligena* or *P*. *cruenta* gene is located, the location along the scaffold, the gene orientation, the protein accession of the sequence if one has been annotated, the Interpro and KOG descriptions, and the conserved domains identified in the Conserved Domain Database (CDD) via blastp or blastx, via the CDD v3.18–55570 PSSMs or NCBI_curated—16212 PSSMs databases. Blastx or blastp analysis was done for each gene or protein sequence using the non-redundant protein sequence database on NCBI, and the top ten hits are indicated with species wherein they are found, description of the sequence, bitscore, E-value, percent sequence identity, and accession. If predicted protein sequences were previously annotated, then reciprocal blastp searches were used to identify homologs of *P*. *fijiensis* sequences, conserved domains were identified in the CDD database using blastp, and blastp analysis was used to identify the top ten hits from the non-redundant protein sequence database on NCBI. If predicted protein sequences were not previously annotated, then putative genes were identified from the *P*. *fuligena* and *P*. *cruenta* genomes using tblastn of *P*. *fijiensis* cluster genes as queries, blastx was used to verify that they were reciprocal best hits, blastx was used to identify conserved domains, and blastx was used to identify the top ten hits from the non-redundant protein sequence database on NCBI. A) The putative biosynthetic cluster for *P*. *fuligena*; B) The putative biosynthetic cluster for *P*. *cruenta*.
(XLSX)

S4 Table Prediction of NRPS domains from *Pseudocercospora* species. For each species, the table indicates NRPS domains predicted by the program PKS/NRPS Analysis Web-site [43], the location of those domains within the predicted protein sequence, the residues within the adenylation domain(s), and the substrate prediction based on the residues within the adenylation domain. A = adenylation domain, PP = thiolation, phosphopantetheine acyl carrier protein domain, C = condensation domain, M = methylation domain,? = unknown domain.
(XLSX)

S5 Table Identification of conserved domain sequences located in regions between previously identified genes. For genome sequences corresponding to putative intergenic regions between previously predicted genes, conserved domain database searches were performed using the blastx algorithm, to identify any additional genes. For each sequence analyzed, the table indicates the species, the genome assembly scaffold, the total length of the scaffold, the position of the putative intergenic region within the scaffold, and conserved domains identified using the conserved domain database, via either the database CDD v3.18 or NCBI_curated.
(XLSX)

S6 Table Mycosphaerellales genome sequences used to identify homologs of *P*. *fijiensis* PKS10-2. The table indicates each species used for tblastn analysis, as well as the NCBI accession of each genome assembly.
(XLSX)
