# Supplementary figures and images for: A polyketide synthase gene cluster required for pathogenicity of Pseudocercospora fijiensis on banana
Source: PLoS One. 2021 Oct 27;16(10):e0258981. doi: 10.1371/journal.pone.0258981 (PMC8550591; doi:10.1371/journal.pone.0258981)

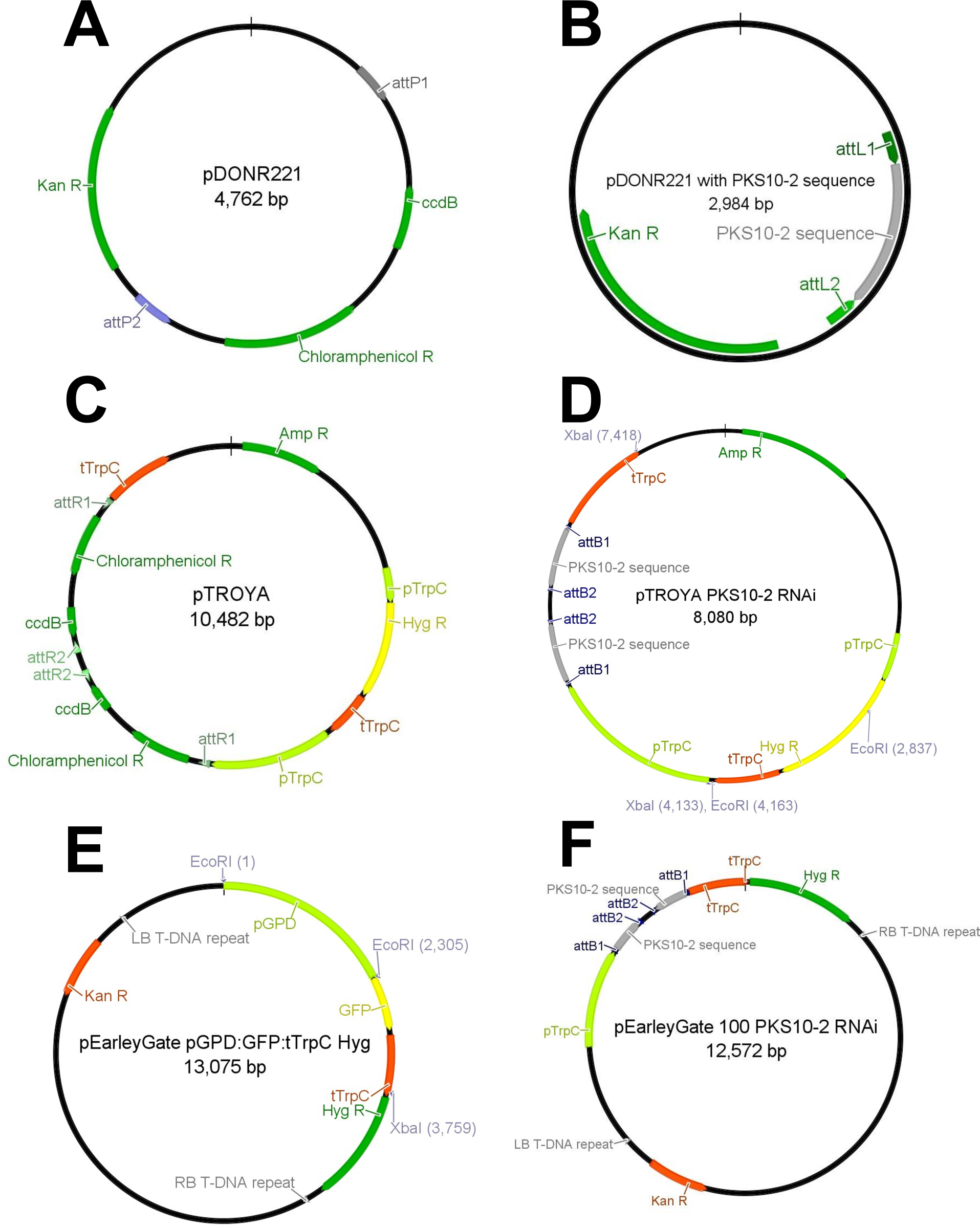

Supplement: S1 Fig — To generate the Agrobacterium mediated transformation-compatible PKS RNAi constructs, a portion of the PKS gene was amplified by PCR with primers that add Gateway attB sites at the ends of the PCR product. A BP reaction was then performed between this PCR product and pDONR221 (A) to generate an entry clone (B) with the PKS sequence contained in the pDONR221 backbone. An LR reaction was then performed with this entry clone and pTROYA (C) to generate an expression clone (D) with a fungal promoter to drive expression of the PKS sequence in inverted repeats, followed by a fungal terminator, in the pTROYA backbone. The PKS RNAi cassette was then transferred from the pTROYA backbone into a modified pEarleyGate 100 plasmid (E) [36] such that the final plasmid in the pEarleyGate 100 backbone contains both the PKS RNAi expression cassette and a hygromycin resistance cassette (F). Plasmid maps were generated in Geneious 6.1.8 (https://www.geneious.com). The figure shows the PKS10-2 silencing constructs; PKS8-2 silencing constructs were generated in the same way. (TIF) [file pone.0258981.s001.tif]
